# *Lactobacillus rhamnosus* GG soluble mediators ameliorate early life stress-induced visceral hypersensitivity and changes in spinal cord gene expression

**DOI:** 10.1042/NS20200007

**Published:** 2020-11-23

**Authors:** Karen-Anne McVey Neufeld, Conall R. Strain, Matteo M. Pusceddu, Rosaline V. Waworuntu, Sarmauli Manurung, Gabriele Gross, Gerry M. Moloney, Alan E. Hoban, Kiera Murphy, Catherine Stanton, Timothy G. Dinan, John F. Cryan, Siobhain M. O'Mahony

**Affiliations:** 1 APC Microbiome Ireland, University College Cork, Cork, Ireland; 2 Teagasc Food Research Centre, Moorepark, Co. Cork; 3 Mead Johnson Pediatric Nutrition Institute, Evansville, IN, USA; 4 Mead Johnson Pediatric Nutrition Institute, Nijmegen, The Netherlands; 5 Department of Psychiatry and Neurobehavioural Science, University College Cork, Cork, Ireland; 6 Department of Anatomy and Neuroscience, University College Cork, Cork, Ireland

**Keywords:** Early life stress, Lactobacillus, Microbiome-Gut-Brain axis, Probiotics, Visceral pain

## Abstract

Visceral hypersensitivity is a hallmark of many functional and stress-related gastrointestinal disorders, and there is growing evidence that the gut microbiota may play a role in its pathophysiology. It has previously been shown that early life stress-induced visceral sensitivity is reduced by various probiotic strains of bacteria (including Lactobacillus rhamnosus GG (LGG)) alone or in combination with prebiotic fibres in rat models. However, the exact mechanisms underpinning such effects remain unresolved. Here, we investigated if soluble mediators derived from LGG can mimic the bacteria’s effects on visceral hypersensitivity and the microbiota–gut–brain axis. Rats were exposed to maternal separation (MS) from postnatal days 2–12. From weaning onwards both non-separated (NS) and MS offspring were provided drinking water with or without supplementation of standardized preparations of the LGG soluble mediators (LSM). Our results show that MS led to increased visceral sensitivity and exaggerated corticosterone plasma levels following restraint stress in adulthood, and both of these effects were ameliorated through LSM supplementation. Differential regulation of various genes in the spinal cord of MS versus NS rats was observed, 41 of which were reversed by LSM supplementation. At the microbiota composition level MS led to changes in beta diversity and abundance of specific bacteria including *parabacteroides*, which were ameliorated by LSM. These findings support probiotic soluble mediators as potential interventions in the reduction of symptoms of visceral hypersensitivity.

## Introduction

The occurrence of stress during critical windows of development has extensive and long-term effects on behavior and physiology, which have been observed both preclinically and clinically [1–6]. Observed alterations include, but are not limited to, changes in behavior, brain and gut function and the intestinal microbial profile [7–10]. Early life stressful events are considered a risk factor for a number of psychiatric and gastrointestinal disorders, some of which are highly comorbid [11], indicating a role for the gut–brain axis in the pathogenesis of these disorders.

Maternal separation (MS) of rat pups is a robust and reliable model of early life stress that induces long-term alterations to behavior, brain neurochemistry and gut physiology [12]. Affected adult offspring demonstrate stress hyperresponsivity, depressive-like behavior, increased visceral sensitivity, intestinal epithelial barrier dysfunction and reduced gut microbial diversity [12,13], and probiotic feedings have been shown to ameliorate some of these stress-induced alterations [14–17]. We have previously shown that the probiotic *Lactobacillus rhamnosus* GG (LGG) has potential beneficial effects in this model of early life stress when administered in the drinking water [18]. MS-induced visceral hypersensitivity to colorectal distension (CRD) is a particularly robust outcome that is sensitive to reversal by a variety of probiotic strains (including LGG) alone or in combination with prebiotic fibres in rat models [10,19–23].

In a different animal model, nociceptive changes induced by chronic stress were partially explained by neuroinflammation in the spinal cord [24]. Specifically, hypersensitivity related to the colorectal region of the gastrointestinal tract is associated with changes in gene expression in the lumbosacral segment of the spinal cord [25,26]. This is due to the fact that the colorectum receives predominant innervation from the lumbosacral spinal cord, via the sacral pelvic nerves [27].

There is a growing emphasis on potential novel probiotic-related therapeutic approaches across different fields of biomedicine [28]. Is it possible that products of probiotic metabolism are involved in biological effects, and therefore could deliver benefits without requiring the presence of the live organism per se. Interestingly, supernatants derived from probiotic LGG have been shown to exert a number of effects including immunomodulatory activity, and feeding neonatal mice these soluble mediators ameliorates inflammatory responses in a model of allergic airway inflammation [29]. Additionally, in a model of short-bowel syndrome, supplementing rats with LGG soluble mediators improved intestinal barrier disruption and inflammation, and in fact was more effective than viable LGG supplementation [30]. While a number of studies have shown effects of soluble mediators derived from LGG and other bacterial strains [31–36] in other cases only live bacterial cells exerted bioactivity on host physiology compared with supernatant [37].

Thus, there is a current interest in better understanding mechanisms of action underlying beneficial effects of soluble mediators of probiotics on gut brain axis function. To this end, we assessed the potential long-term benefits of feeding a specific preparation of soluble mediators derived from the probiotic LGG to rats that had been previously subjected to early life MS in terms of visceral pain sensitivity, and whether this observation could partially be explained by spinal gene expression and modulation of the gut microbiota profile.

## Experimental

### Animals

Fifteen pairs of male and female Sprague-Dawley rats were purchased from Harlan, U.K. and habituated to the Preclinical Research Facility, University College Cork for 1 week. Rats were mated and all 15 females became pregnant. Animals were kept under controlled conditions (21 ± 1°C) on a 12-h light/dark cycle (lights on 7:00 am) and fed *ad libitum*. Dams with litters and later weaned offspring were maintained in animal cages, dimensions (42 × 25 × 13 cm). All experiments were conducted in accordance with the European Directive 2010/63/EEC, the Recommendation of the S.I No 543 of 2012, and approved by the Animal Experimentation Ethics Committee of the University College Cork.

Please see timing of each experimental procedure on Supplementary Figure S1.

### Maternal separation

Day of birth was designated as postnatal day (PND) 0. MS protocol was adapted from that described in [38]. Briefly, at PND0 litters were randomly assigned to undergo MS or to remain as non-separated (NS) controls. At PND2 the litters assigned to MS were removed from the main colony room to an adjacent room where the entire litter was placed into a separate, smaller cage maintained at 30–33°C. Mothers were returned to the home cage and the main colony room so as to prevent communication via ultrasonic vocalization. After a 3 h separation the mothers were again brought into the adjacent room and reunited with their respective litters. Control litters were left undisturbed in the mothers’ home cage except for routine cage cleaning performed once a week. This procedure was repeated from PND2 to PND12 inclusive. The period of separation was carried out between 9:00 am and 12:00 pm daily.

### Dietary intervention and preparations

At PND21 pups were weaned, females were culled and only male rats were used for the remainder of the experiment. Male offspring were randomly group-housed by intervention (three per cage) with no littermates housed together. NS and MS male offspring were randomized into different groups and provided drinking water with or without supplementation of LGG soluble mediators (LSM) from 3 weeks of age until approximately 13 weeks old (end of the experiment). This yielded four separate groups. LSM was produced as previously described (Wu et al., 2018): LGG was cultured under standardized conditions, and spent medium was collected at a defined time point during the late exponential growth phase. This supernatant was desalted, sterile filtered and lyophilized. This specific preparation protocol intended to remove lactic acid that is a nonspecific product of lactic acid bacteria fermentation, as well as any bacterial cells. Reconstituted material was daily freshly prepared in de-ionised drinking water with mixing of the drinking bottle 8 to 10 h after first placing it in the cage. The preparation was at a dose equivalent to 1.3–4.3 × 10^8^ CFU viable LGG/animal/day) of the original LGG culture. All animals received a modified American Institute of Nutrition 93-G control diet (Envigo, WI U.S.A.). Interventions continued throughout behavioural testing. Body weight was measured weekly, water intake daily, and food intake 3 times/week. Twelve animals/group were assessed throughout.

### Colorectal distension (CRD)

CRD occurred when the animals were 11 weeks of age and was carried out during the light cycle of the day. This test was conducted as previously described [39]. Animals were fasted for 24 h and then lightly anaesthetised using isofluorane. A 6 cm polyethylene balloon with a connecting catheter was inserted in the distal colon 1 cm proximal to the anus and fixed to the tail with tape to avoid displacement. Animals were allowed to recover from anaesthesia for at least 15 min before beginning colorectal distension. A customized barostat was used to control balloon inflation and pressure during colorectal distension. The distension paradigm used was an ascending phasic distension from 0 to 80 mmHg. A trained and blinded observer scored each rat for the threshold, or pressure when the first pain behaviour was noted as well as the number of pain behaviours exhibited by each animal.

### Determination of plasma corticosterone

At 12 weeks of age and one week following CRD, animals were removed from home cages at 0830 h and placed in clear Perspex rat restrainers with tails exposed. The tip of each rat’s tail was then cut at an angle with a scalpel blade and blood collected in a 1.5 ml collection tube (approximately 200 μls/time point). The initial blood collection was marked T0 and corresponded to a baseline collection. Animals were then singly housed in restrainers for 30 min. Following the stressor, the animal was removed from the restrainer and a T30 blood collection occurred. The animal was placed back into the home cage after a brief recovery period. Blood was stored on wet ice until spun in a centrifuge and the plasma collected. Plasma was stored in −80°C freezer until an ELISA assay for corticosterone was performed.

### Tissue extraction and RNA sequencing

At 13 weeks of age all animals were culled and samples collected. RNA from rat lumbosacral spinal cord was extracted using commercially available mirVana™ Total Rna Extraction Kit (Ambion/Life Technologies) and DNase treated (Turbo DNA-free, Ambion/Life Technologies) according to manufacturer’s protocol. RNA was high quality with an RNA integrity number value for all samples above 8 (Bioanalyzer, Agilent). Spinal cord samples were then randomly pooled within each group by combining equal amounts of RNA from two to three animals resulting in a final four samples per group sent for RNA sequencing at GATC (Konstanz, Germany), where library preparation, paired-end sequencing (2 × 125 bp) and Fastq-file generation was conducted on an Illumina HiSeq sequencer.

### Differential gene expression and GO-term enrichment analyses

Ensembl release 78 was used for read counting for each gene using HTSeq-Count (v0.6.0) with the following non-default parameters: -s: no; -r: pos; -q –f bam –m intersection-nonempty. Differential gene expression was determined using the DESeq2 R-package (v1.6.2) with default parameters on pairwise comparisons of all possible group combinations. An adjusted *P*-value ≤ 0.1 (Benjamini–Hochberg method) was considered significantly differentially regulated [40–42]. Differentially expressed genes were then analysed for enrichment of GO-Terms using the DAVID Bioinformatic Resources (v6.8).

### Microbiota analysis

Fresh caecal samples were collected from individual rats at the end of the study. At least 20 mg of fresh caecal material were placed in a microcentrifuge tube and kept on ice until storage at −80°C. DNA was extracted using the DNA Fast Stool DNA Extraction Kit (Qiagen) using the protocol for Gram positive bacteria and including an additional bead beating step at the beginning of the procedure. The microbiota composition of the samples was established by amplicon sequencing of a ∼380 bp fragment of the V3-V4 hypervariable region of the bacterial 16S rRNA gene as outlined in the Illumina 16S Metagenomic Sequencing Library preparation guide (Illumina). Amplicons were quantified, normalised and pooled using the Qubit® dsDNA HS Assay Kit (Life Technologies). Samples were sequenced on the MiSeq sequencing platform at Clinical Microbiomics, using a 2 × 300 cycle kit, following standard Illumina guidelines.

### Microbiota bioinformatics

Forward and reverse reads were merged using USEARCH requiring a merged minimum length of 300 bp, maximum 600 bp and minimum 100 bp overlap. A maximum of one expected error was allowed per read. Primers were trimmed, discarding any sequences that do not have a perfect match to both forward and reverse primers. Sequences were dereplicated, discarding clusters smaller than 5 and clustered at 97% sequence similarity using USEARCH [43]. Suspected chimeras were removed using UCHIME [44]. Taxonomy was assigned to each operational taxonomic unit (OTU) using the RDP Classifier [45]. To estimate alpha diversity, the data set was rarefied to the same number of sequences and the Shannon diversity index was calculated using the diversity function from the vegan package in R [46]. Beta diversity was also calculated in R using Bray–Curtis distances and Principal Coordinate Analysis (PCoA) was performed using the vegan package.

### Statistical analysis

Data was analysed using a repeated measures two-way ANOVA, a two-way ANOVA and LSD’s *post-hoc* test where appropriate using the statistical software package SPSS 22.0 (IBM). A *P*-value of 0.05 was selected as the threshold of statistical significance. The rank Kruskal–Wallis test was used for comparison of taxa followed by Dunn’s test with false discovery rate *P*-value adjustment for multiple comparisons.

The Mann *U* Whitney test was used to assess for differences between the various groups. The Benjamini–Hochberg for false discovery rate was used to calculate *q* (*Q* was set at 10%). A two-way ANOVA was carried out on alpha diversity indices for intervention and separation and the interaction between intervention and separation and the independent *t* test was employed for pairwise comparisons. Beta diversity was compared between groups using the adonis function in the R vegan package at the genus level, using Bray–Curtis as distance measurement.

## Results

### Effect of LSM supplementation on animal weights and food and water intake

A repeated measures two-way ANOVA showed that all rats gained weight over time (F_(8,352)_ = 4960, *P*<0.0001) and post hoc analyses showed no specific differences between groups at any timepoint (Supplementary Figure S2A).

A repeated measures two-way ANOVA showed that all animals increased their food intake over time (F_(6,72)_ = 471.8, *P*<0.0001) and a main effect for intervention on food intake (F_(3,12)_ = 5.38, *P*=0.01), but again post hoc analyses showed no specific differences between groups in terms of amount of food ingested at a particular timepoint (Supplementary Figure S2B).

There was a main effect for fluid intake over time (F_(7,84)_ = 131, *P*<0.0001), but no main effect for LSM on fluid intake (F_(3,12)_ = 2.73, *P*=0.09) and no specific differences between groups in terms of fluid intake at a particular timepoint (Supplementary Figure S2C).

### Effect of feeding LSM on visceral sensitivity

**Figure 1:**
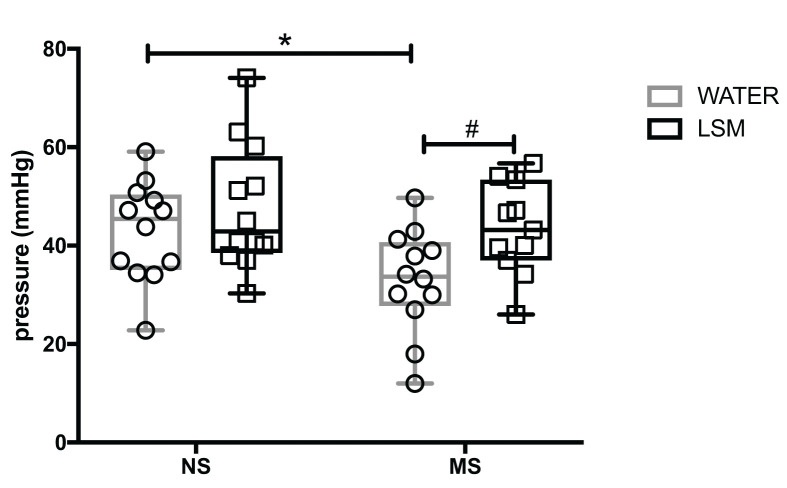
The threshold of visceral sensitivity in colorectal distension Early life stress caused lower pain thresholds as measured by CRD in MS versus NS control rats that was ameliorated by supplementation with LSM. *P=0.04 versus NS control; #*P*=0.001 versus MS control. Data are represented as mean ± SEM, *n*=12/grp.

A significant main effect for the intervention was observed in the CRD test for visceral sensitivity (F_(1,44)_ = 7.1, *P*=0.01), but no main effect for early life stress (F_(1,44)_ = 2.66, *P*=0.11). There was no significant interaction (F_(1,44)_ = 0.8267, *P*=0.3683). Fisher’s LSD *post hoc* showed a specific difference between MS control rats versus NS control (*P*=0.04) with MS rats showing lower pain thresholds. There was also a significant difference between MS LSM rats versus MS controls (*P*=0.01) in that LSM feeding resulted in higher pain thresholds in the early life stressed groups (see Figure 1). There was no difference between MS control and MS rats fed bacterial free unconditioned medium (data not shown) nor was there any differences noted for the number of pain behaviors.

### Effects of feeding LSM on stress responsivity

**Figure 2:**
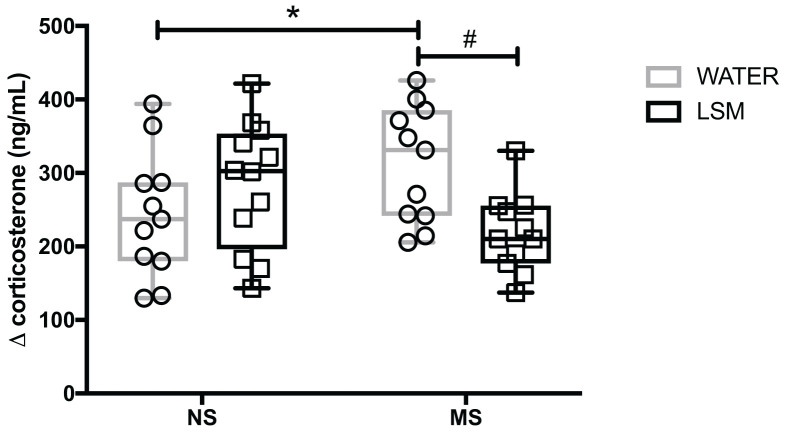
Stress reactivity as measured by plasma corticosterone Early life stress caused a significant increase in plasma corticosterone values following a 30 min restraint stress, which was attenuated by LSM supplementation. *P=0.04 versus NS control; #*P*=0.007 versus MS control. Data represented as mean ± SEM, *n*=12/grp.

Measures of plasma corticosterone were made for both the T0 (baseline) and T30 (stressed) timepoints and a **Δ**corticosterone value was calculated from these for each animal. A significant early life and intervention interaction was observed (F_(1,41)_ = 8.49, *P*=0.006), but no main effect for either early life stress (F_(1,41)_ = 0.006, *P*=0.93) or supplementation (F_(1,41)_ = 1.32, *P*=0.26). Fisher’s LSD post hoc showed a significantly higher **Δ**corticosterone for MS versus NS controls (*P*=0.04) that was attenuated after LSM intervention (*P*=0.007) (see Figure 2).

### Effects of feeding LSM on mRNA expression in the spinal cord

**Figure 3:**
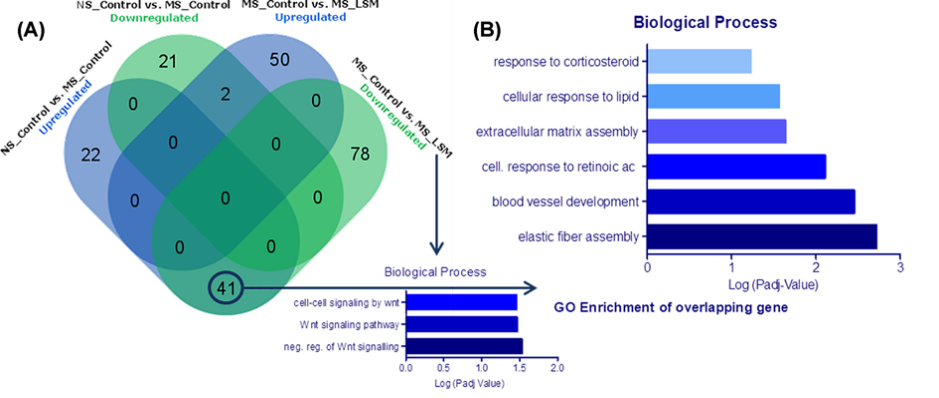
mRNA expression in spinal cord and functional enrichment analysis of the overlapping genes showed that 12 of these were associated with pain, stress or both (**A**) Numbers of gene differentially expressed between groups including GO enrichment of overlapping genes particularly associated with the Wnt signaling. (**B**) Biological processes linked to differentially expressed genes. Of the 63 spinal cord genes that were up-regulated by MS, 41 of these were down-regulated with LSM administration. Spinal cord samples from three animals within each group were randomly pooled resulting in a final four samples per group sent for RNA sequencing.

Unbiased deep sequencing of mRNA was carried out on spinal cord tissue. Analysis of gene expression by pairwise comparisons revealed a number of differentially regulated genes across experimental groups (*P*<0.1). First, when comparing mRNA expression profile of NS and MS control animals a total of 86 differentially expressed genes were identified (23 down-regulated and 63 up-regulated) (Figure 3A). Next, we investigated the impact of LSM supplementation on spinal cord gene expression in MS rats compared with MS control animals. A total of 171 genes were identified (119 down-regulated and 52 up-regulated). Interestingly, we found 41 genes that were up-regulated in MS control animals compared with NS, and were down-regulated after administration of LSM (Figure 3A and Table 1). We also determined that amongst the total down-regulated genes (78+41 = 119) in MS animals after LSM supplementation, there was a significant enrichment in Wnt family of signaling peptides which are key players in pain (Dkk2, Tcf7, Nxn, Lef1, Nfatc4, Cdh1, Col1a1, Apcdd1 and Cpz) (Figure 3A). Within this subset of overlapping genes, we investigated biological function linked to these genes. GO analysis showed significant enrichment for biological processes specifically associated with stress (Figure 3B). All gene changes are now included on volcano plots with the horizontal dashed line being fold change and vertical dashed line is 0.1 which is the cut off for a *P* value when it is logged (Supplementary Figure S3A,B).

**Table 1:** Spinal cord genes noted to be up-regulated due to maternal separation and down-regulated by the administration of LSM

Description	Associated gene name	NS versus MS Log2 (fold change)	MS versus MS + LSM Log2 (fold change)
Annexin A1	Anxa1	−0.89775	−0.822068
Annexin A8	Anxa8	−1.23281	−2.17727
Asialoglycoprotein receptor 2	Asgr2	−0.51856	−0.481867
Biglycan	Bgn	−0.26825	−0.419479
Complement component 7	C7	−0.46183	−0.467474
Cyclin F	Ccnf	−0.69414	−0.638514
Cholinergic receptor nicotinic beta 3 subunit	Chrnb3	−0.81698	−0.819548
Collagen, type XVIII, alpha 1	Col18a1	−0.38027	−0.549118
Collagen, type I, alpha 1	Col1a1	−0.70161	−0.904032
Collagen, type VI, alpha 1	Col6a1	−0.40196	−0.590088
Collagen, type VI, alpha 2	Col6a2	−0.43194	−0.516948
Carboxypeptidase X (M14 family), member 2	Cpxm2	−0.38027	−0.907097
Carboxypeptidase Z	Cpz	−0.9267	−0.823511
Cytokine like 1	Cytl1	−0.73819	−1.21628
Elastin	Eln	−0.78315	−0.946259
Endothelial cell-specific molecule 1	Esm1	−1.14174	−1.53702
Filamin A	Flna	−0.29314	−0.716614
Glutathione S-Transferase M2	Gstm2	−0.6405	−0.43816
Hydroxycarboxylic acid receptor 1	Hcar1	−0.8035	−0.66941
Interferon induced transmembrane protein 2	Ifitm2	−0.56305	−0.39145
Insulin-like growth factor binding protein 3	Igfbp3	−0.80728	−0.78094
Insulin-like growth factor binding protein 4	Igfbp4	−0.47974	−0.50599
Keratin 10	Krt10	−0.94947	−0.43816
Keratin 13	Krt13	−3.88679	−2.58248
Lectin, galactoside-binding, soluble, 7	Lgals7	−2.47941	−2.52878
Leukocyte immunoglobulin-like receptor, subfamily B (with TM and ITIM domains), member 3-like	Lilrb3l	−1.26735	−0.87364
Lysyl oxidase	Lox	−0.47596	−1.04757
Microtubule-associated protein, RP/EB family, member 2	Mapre2	−0.60424	−0.80124
Mannose receptor, C type 1	Mrc1	−0.46494	−0.56762
Myosin, heavy chain 11, smooth muscle	Myh11	−0.46892	−0.33294
Pappalysin 2	Pappa2	−0.76301	−1.38181
Procollagen C-endopeptidase enhancer	Pcolce	−0.63158	−1.34621
Periostin	Postn	−0.87747	−0.41055
Polymerase I and transcript release factor	Ptrf	−0.31889	−0.36173
RT1 class II, locus Bb	RT1-Bb		−0.43733
Serpin family F member 1	Serpinf1	−0.71682	−0.52703
Thrombospondin 2	Thbs2	−0.29513	−0.29096

### Effects of feeding LSM on caecal microbiota

#### Alpha diversity

**Figure 4:**
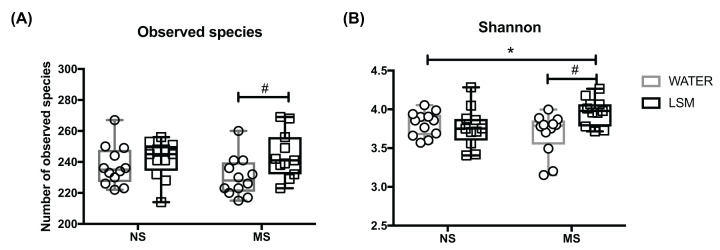
Alpha diversity of the caecal microbiome (**A**) Observed species/ richness was increased on the MS LSM group. (**B**) Shannon index/species diversity was increased in the MS LSM group also. *P<0.05 versus MS water group; **P<0.01 versus MS water group.

Alpha diversity of the samples was measured by observed species and Shannon diversity. The observed species index measures the number of different bacterial species per sample that is defined as ‘richness’. However, regarding diversity, not only the qualitative amount of species, but also the abundance of the species must be taken into account. The relative abundances of the different species making up the samples’ richness are defined as ‘evenness’. The Shannon-diversity index relates both, OTU richness and evenness. A two-way ANOVA for LSM and stress and the interaction between these on alpha diversity indices was carried out. There was a significant effect of LSM on number of observed species F(1,43) = 5.57, *P*=0.02 (Figure 4A), with no significant interactions between stress and LSM. There was a significant interaction between stress and LSM for Shannon F(1,43) = 6.97, *P*=0.02. There was no significant effect of stress on the alpha diversity indexes (Figure 4B).

### Taxonomic analysis

#### NS control versus MS control

The effect of MS was assessed at the three taxonomic levels by comparing NS unsupplemented control rats with MS control rats. At the phylum level, there was a decrease in the relative abundance of Proteobacteria in MS rats (*P*=0.04; *q*=0.01), while at the family level, Cryomorphaceae (*P*=0.02; *q*=0.003) abundances were significantly lower and Prevotellaceae (*P*=0.02; *q*=0.005) significantly higher in MS animals compared with NS. MS significantly lowered the relative abundance of 6 genera (Clostridium cluster XIVa (*P*=0.002; *q*=0.001); Bilophia (*P*=0.006; *q*=0.002); Wandonia (*P*=0.02; *q*=0.004); Acetivibrio (*P*=0.03; *q*=0.005); Parabacteroides (*P*=0.04; *q*=0.006) and Anaerosinus (*P*=0.04; *q*=0.007) and increased Alloprevotella (*P*=0.02; *q*=0.003) populations (see Table 1 and Supplementary Figure S4).

#### NS control versus NS LSM

LSM supplemented NS animals had significantly lower Proteobacteria (*P*=0.01; *q*=0.01) and Desulfovibrionaceae (*P*=0.03; *q*=0.005) relative abundances compared with NS control rats. While relative abundance of Ruminococcaceae was significantly increased in LSM NS animals (*P*=0.002; *q*=0.003) as was Faecalibacterium (*P*=0.03; *q*=0.002). Acetitomaculum (*P*=0.03; *q*=0.001) relative abundances was noted to be lower than in NS control rats (see Table 1 and Supplementary Figure S4).

#### MS control versus MS LSM

In MS rats, LSM treated animals had significantly greater Peptostreptococcaceae (*P*=0.006; *q*=0.006), Barnesiella (*P*=0.03; *q*=0.006), Parabacteroides (*P*=0.019; *q*=0.005), Oribacterium (*P*=0.02; *q*=0.004) and Clostridium cluster XI (*P*=0.006; *q*=0.003) relative abundances compared with MS control rats. In contrast, relative abundance of Sutterellaceae (*P*=0.004; *q*=0.003), Tannerella (*P*<0.001; *q*=0.001) and Parasutterella (*P*=0.004; *q*=0.002) in LSM MS rats was lower compared with control MS animals (see Table 2 and Supplementary Figure S4).

**Table 2:** The significant differences seen in the caecal microbiota of all groups at phyla, family and genus level

NS Control versus MS Control				
Taxa	NS Control	MS Control	*P* value	*q* value
**Phylum**				
*Proteobacteria*	2.49%	1.83%	0.039	0.0125
**Family**				
*Cryomorphaceae*	0.04%	0.01%	0.02	0.0026
*Prevotellaceae*	0.97%	2.22%	0.024	0.0053
**Genus**				
*Clostridium cluster XIVa*	7.66%	3.60%	0.002	0.0010
*Bilophia*	0.22%	0.07%	0.006	0.0019
*Alloprevotella*	0.93%	2.21%	0.02	0.0029
*Wandonia*	0.04%	0.01%	0.02	0.0039
*Acetivibrio*	0.03%	0.11%	0.028	0.0049
*Parabacteroides*	0.58%	0.44%	0.039	0.0058
*Anaerosinus*	0.04%	0.02%	0.039	0.0068

NS control versus NS LSM				
Taxa	NS Control	NS LSM	*P* value	*q* value
**Phylum**				
*Proteobacteria*	2.49%	1.78%	0.017	0.0125
**Family**				
*Ruminococcaceae*	22.80%	31.29%	0.002	0.0026
*Desulfovibrionaceae*	1.95%	1.34%	0.033	0.0053
**Genus**				
*Faecalibacterium*	3.95%	9.13%	0.028	0.0019
*Acetitomaculum*	0.03%	0.21%	0.033	0.0010

MS Control versus MS LSM				
Taxa	MS Water	MS LSM	*P* value	*q* value
**Family**				
*Sutterellaceae*	0.09%	0.01%	0.004	0.0026
*Peptostreptococcaceae*	1.84%	3.37%	0.006	0.0053
**Genus**				
*Tannerella*	18.50%	9.32%	0.001	0.0010
*Parasutterella*	0.09%	0.01%	0.004	0.0019
*Clostridium cluster XI*	1.84%	3.37%	0.006	0.0029
*Oribacterium*	0.18%	0.67%	0.016	0.0039
*Parabacteroides*	0.44%	0.62%	0.019	0.0049
*Barnesiella*	19.53%	24.02%	0.032	0.0058

% refers to percentage of relative abundance of bacteria. *Q* value is the adjusted *P* value for false discovery rates

### Beta diversity of caecal microbiome

Bray–Curtis plots were generated from genus files. The Adonis Permanova test was carried out to assess whether caecal microbiome had significantly dissimilar beta diversity due to early life stress or administration of LSM.

**Figure 5:**
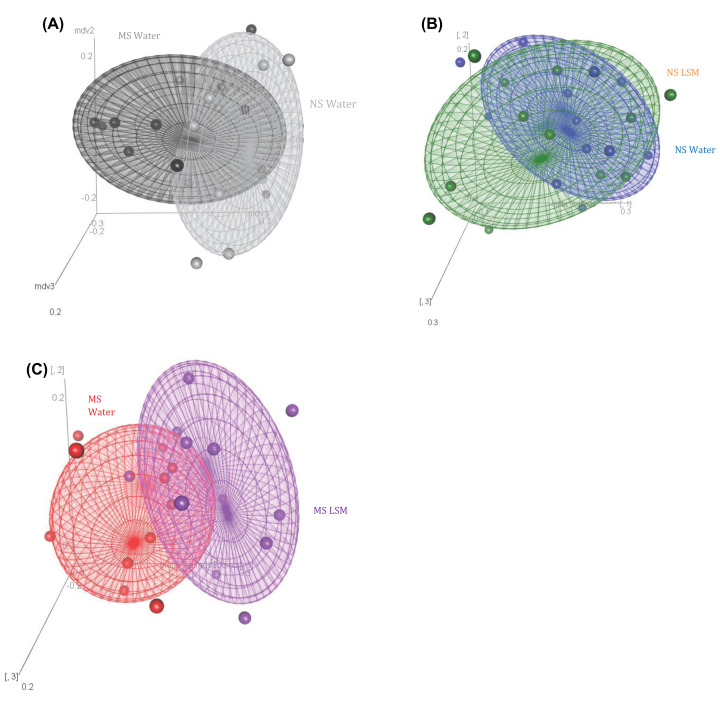
Beta diversity of the caecal microbiome (**A**) Bray–Curtis plot of NS Water and MS Water rats caecal microbiomes shows significant impact of early life stress on beta diversity. (**B**) NS LSM and NS Water rats caecal microbiomes with no differences in beta diversity observed (overlapping area). (**C**) Bray–Curtis plot of MS LSM and MS Water rats caecal microbiomes shows significant impact of LSM on beta diversity in the stressed group.

Early life stress significantly impacted on the beta diversity, seen as differences between the MS control group receiving water only and the NS control group (*P*<0.05). There was no significant difference between the NS groups receiving LSM or water (*P*=0.18). In contrast, MS LSM rats had a significantly dissimilar caecal microbiome to control counterparts (*P*=0.001) (see Figure 5).

## Discussion

There is a growing interest on the potential of probiotic strains to ameliorate the effects of stress. Within the mechanisms underpinning such effects, the production of neuroactive metabolites is emerging as a key player [47]. The present study implicates soluble mediators derived from the probiotic LGG to exert beneficial effects on stress induced visceral hypersensitivity.

In the present study, as previously shown early life stress led to an increased sensitivity to colorectal distension [26,48–51]. Interestingly, we noted that feeding with LSM from weaning onwards resulted in an attenuation of this visceral hypersensitivity, indicating that this probiotic-derived intervention ameliorates early-life stress-induced dysfunction. Given the co-morbidity between gastrointestinal and stress-related disorders we also investigated the impact of LSM on hypothalamic–pituitary–adrenal (HPA) axis responsivity by measuring the response to restraint stress. We noted that an increased HPA axis activity induced by early life stress was also amenable to reversal by LSM.

We have previously reported that early life MS in rats results in specific changes in gene expression in the lumbosacral spinal cord that were associated with increased visceral pain sensitivity [25,26]. Here using RNA sequencing we expand on our previous data to demonstrate that the expression levels of a total of 86 genes differed between the NS and MS groups. Of interest, 63 of these genes were up-regulated as a result of early life MS and from this subset, expression levels were reversed in 41 of these after LSM administration in MS animals. These 41 overlapping genes were further examined by a functional enrichment analysis and 12 of these were found to be associated with pain circuitry, stress, or both. These findings of changes in spinal cord gene expression are thus consistent with both the CRD and HPA results we observed. One of the genes found to be up-regulated by MS and then down-regulated following LSM intervention coded for the protein annexin A1. Annexin A1 is found in abundance in cells involved in neuroendocrine communication, and its synthesis and release is dependent on glucocorticoids. It is also thought to play a significant role in the perinatal programming of the HPA axis [52]. Of the total down-regulated genes seen in MS animals after LSM supplementation, there was a significant involvement in Wnt signaling. Previous studies has indicated a role for WNT signaling in a rat model of neuropathic pain where a rapid-onset and long-lasting expression of WNTs in the primary sensory neurons, the spinal dorsal horn neurons, and astrocytes were noted [53]. Moreover, spinal blockade of WNT signaling pathways prevented the induction of neuropathic pain, the accompanying neurochemical changes and inflammation without affecting normal pain sensitivity and locomotor activity [53]. While further research is needed to better understand the mechanisms regulating the molecular interactions among stress, visceral sensitivity and Wnt signaling, these pathways with regard to the brain–gut axis communication may provide a novel mechanistic insight and effective approach into the possibility to ameliorate the impact of early life stress.

At the microbiota level, alpha diversity of the caecal samples was measured by observed species and Shannon diversity. While early life stress did not induce changes in alpha diversity per se, LSM increased both richness and evenness of the microbiota in the rats exposed to the early life stress perhaps indicating that the microbiota population in the MS rats was more amenable to change.

Beta diversity was assessed using Bray–Curtis dissimilarity based on abundance level and we noted that the NS and MS control groups had different species abundances. Furthermore, we also noted that the MS group receiving LSM group when compared with its control had a different beta diversity. This highlights the fact that both early life stress and LSM induce significant changes to the microbiota at a species abundance level. Furthermore, the impact of LSM on specific caecal microbial populations was not consistent between MS and NS animals suggesting that the effect of LSM on the microbiome could be through altering signaling rather than a direct metabolic/antimicrobial effect. MS altered the microbiome at various taxonomic levels, notably significantly reduced clostridium cluster XIVa relative abundances at the genus level. The clostridium cluster XIVa contains many butyrate producing species and are considered important microbial mediators in gut health and homeostasis [54]. Many species belonging to this cluster have putative anti-inflammatory activities including induction of colonic regulatory T cells [55]. Furthermore, clostridium cluster XIVa have been shown to be reduced in several disease states such as Crohns disease [56] and cystic fibrosis [57].

Drilling down into the microbiota data confirms that LSM administration led to a significantly different microbiota profile yet was unable to reverse the effects of early life stress per se. LSM had a differential impact on the gut microbiota of the NS and MS rats. More specifically in the MS rats, LSM increased the abundance of Barnesiella species. Barnesiella is a genus that has been shown to protect the gut against infection in rats and may be a key regulator of gut homeostasis [58]. LSM also significantly reduced the abundance of Tannerella species. Tannerella species are considered a major oral pathogen [59] and its presence has been linked to increased risk of esophageal cancer [60]. Of particular note is the decrease of the genus Parabacteroides in the MS compared with the NS control group that was reversed by LSM supplementation. Some species within this genus, such as Parabacteroides distasonis has been shown to significantly decrease the severity of gastrointestinal inflammation in murine models of acute and chronic colitis induced by dextran sulphate sodium [61] and also to be negatively correlated with multiple sclerosis, non-alcoholic fatty liver disease and obesity [62,63]. Furthermore, *in vitro* cultivation of Parabacteroides distasonis demonstrated its capacity to transform bile acids and production of succinate [64]. While more mechanistic investigations are necessary, it is possible that changes in the abundance of Parabacteroides may play a role in the beneficial impact of dietary interventions on behavior. This is in line with our previous findings related to the effects of a blend of prebiotics (polydextrose/galacto-oligosaccharide prebiotic blend) positively influencing behaviour and brain gene expression while also increasing the abundance of parabacteroides [65].

The significant and differential increase in Faecalibacterium genera observed NS animals supplemented with LSM compared with NS control animals are also notable as this genera is attributed to have anti-inflammatory properties [66] including antinociceptive effects in anti-inflammatory disease models [37] and has been shown to be protective against pathological gut inflammation in a murine model [67]. LSM was also found to increase Ruminococcaceae populations, another family noted for butyrate production and therefore may play a role in promoting gut health [68].

Taken together, these results suggest that LSM ameliorates stress induced visceral hypersensitivity, can promote gut microbiome diversity in rats that have undergone MS stress and can impact gut microbial composition as well as spinal cord gene expression. In this model, the effects of LSM have not been dependent on administration of viable probiotic bacteria but might have been mediated by LGG metabolites and fermentation products present in the preparation such as short-chain fatty acids, bacteriocins, or immunomodulatory components of LGG such as toll-like receptor ligands. Besides potential direct effects on the host, these might have also exerted indirect effects by influencing the abundance of bacterial species in the intestine. Further studies are needed to address the exact mechanisms. Overall, these findings intriguingly support probiotic soluble mediators as potential interventions in the management of stress induced gastrointestinal symptoms or disorders.

## Supplementary Material

Supplementary Figures S1-S4

## Data Availability

All supporting are included in the main article and its supplementary files.

## Abbreviations

CRD: colorectal distension
HPA: hypothalamic–pituitary–adrenal
LGG: Lactobacillus rhamnosus GG
LSM: LGG soluble mediators
MS: maternal separation
NS: non-separated
PND: postnatal day
