# Supplementary material for: Lactobacillus rhamnosus GG soluble mediators ameliorate early life stress-induced visceral hypersensitivity and changes in spinal cord gene expression
Source: Neuronal Signal. 2020 Nov 23;4(4):NS20200007. doi: 10.1042/NS20200007 (PMC7726314; doi:10.1042/NS20200007)

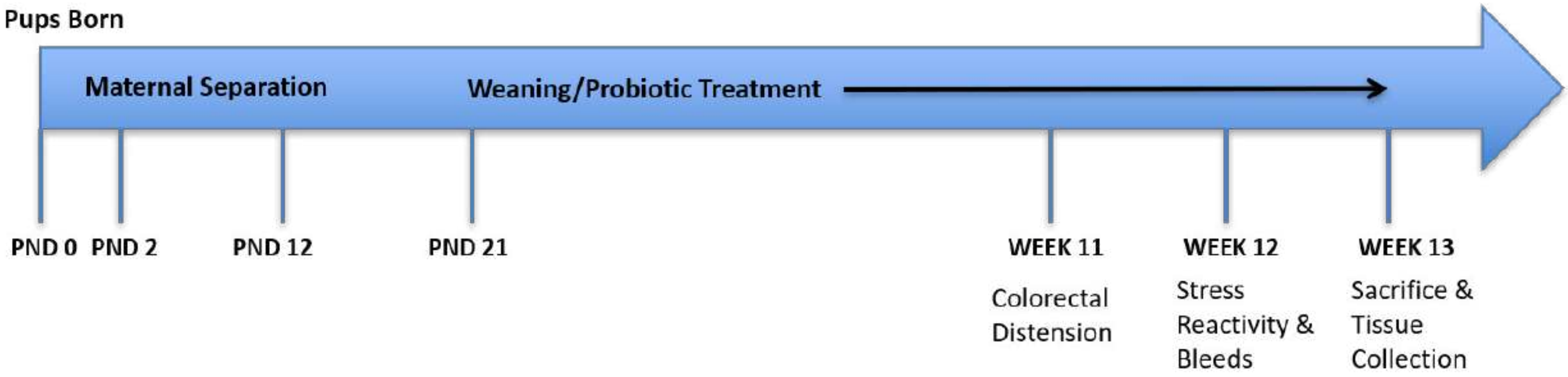

Figure S1: The timeline of experimental procedures. PND=postnatal day.

Figure S2: Early life or intervention caused no differences in weight gain, food intake or fluid intake (A) No differences were observed in animal weights across groups over the experimental period, (B) in dietary intake or in (C) fluid intake.

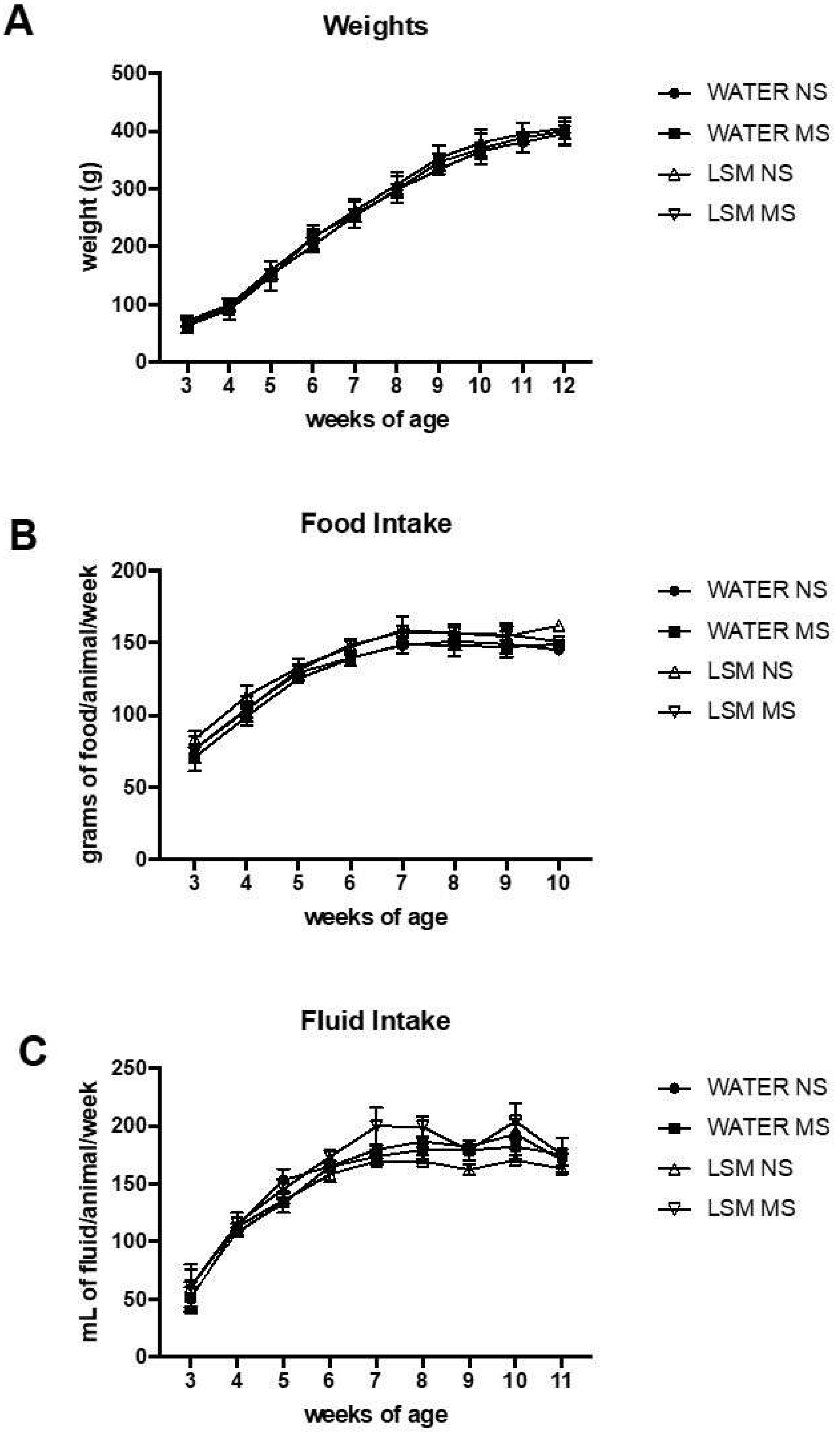

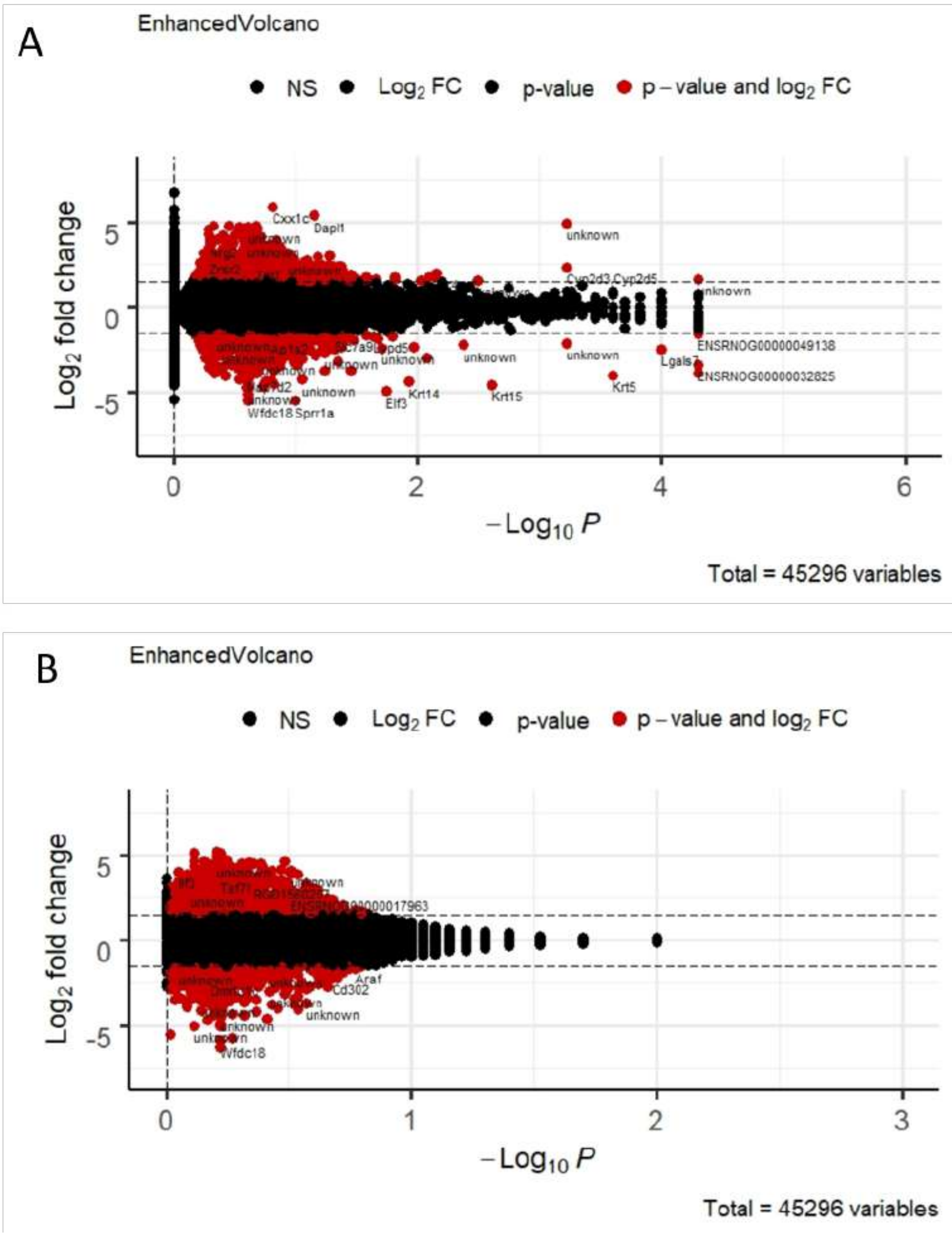

<u>Figure S3: Volcano plots of all genes representing the fold change and p values. The horizontal dashed line is fold change and the vertical dashed line is 0.1 which is the cut off for a p value when it is logged.</u>

Figure S4: Graphical representation of A. the phyla B. the top 10 most abundant families C. the top 10 most abundant genera of the caecal microbiome. Lower abundant family and genera were combined into "other".

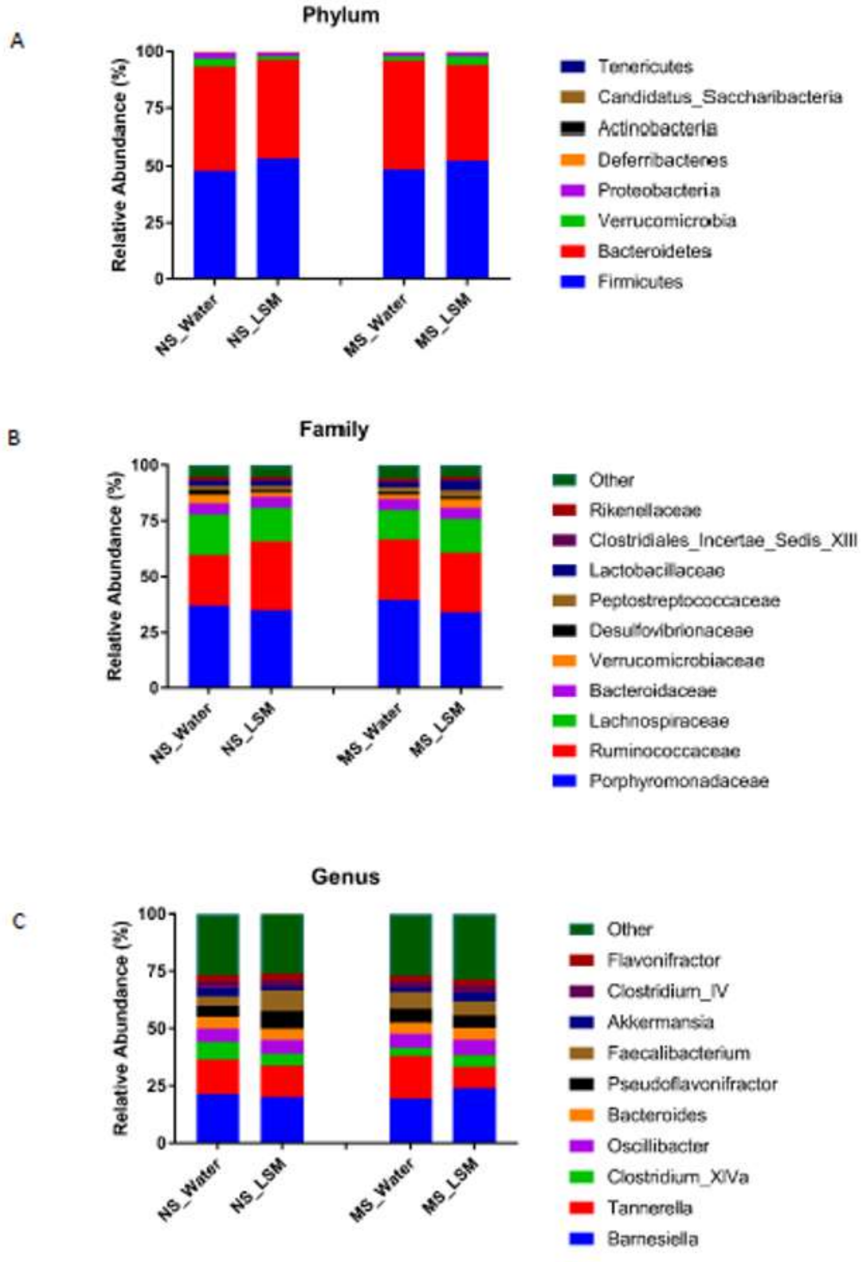

Supplement: Supplementary Figures S1-S4 [file NS-2020-0007_supp.pdf]
